# Optimal self-assembly of lipid nanoparticles (LNP) in a ring micromixer

**DOI:** 10.1038/s41598-022-13112-5

**Published:** 2022-06-08

**Authors:** Manon Ripoll, Elian Martin, Mathilde Enot, Oscar Robbe, Chiara Rapisarda, Marie-Claire Nicolai, Aurélie Deliot, Patrick Tabeling, Jean-René Authelin, Mostafa Nakach, Pierre Wils

**Affiliations:** 1 BioDPD Department, SANOFI, 13 Quai Jules Guesde, 94400 Vitry-sur-Seine, France; 2 Microfluidics, MEMS, Nanostructures Laboratory, CNRS Chimie Biologie Innovation (CBI), UMR 8231, Institut Pierre Gilles de Gennes (IPGG), ESPCI Paris, PSL Research University, 6 rue Jean Calvin, 75005 Paris, France; 3 REI Department, SANOFI Pasteur, 1541 Av. Marcel Mérieux, 69280 Marcy-L’Étoile, France

**Keywords:** Biochemistry, Biotechnology

## Abstract

Lipid nanoparticles (LNPs) for RNA and DNA delivery have attracted considerable attention for their ability to treat a broad range of diseases and to vectorize mRNA for COVID vaccines. LNPs are produced by mixing biomolecules and lipids, which self-assemble to form the desired structure. In this domain, microfluidics shows clear advantages: high mixing quality, low-stress conditions, and fast preparation. Studies of LNPs produced in micromixers have revealed, in certain ranges of flow rates, a degradation in performance in terms of size, monodispersity and encapsulation efficiency. In this study, we focus on the ring micromixer, which is well adapted to high throughput. We reveal three regimes, side-by-side, transitional and highly mixed, that control the mixing performance of the device. Furthermore, using cryo-TEM and biochemical analysis, we show that the mixing performances are strongly correlated to the characteristics of the LNPs we produce. We emphasize the importance of the flow-rate ratio and propose a physical criterion based on the onset of temporal instabilities for producing LNPs with optimal characteristics in terms of geometry, monodispersity and encapsulation yield. These criteria are generally applicable.

## Introduction

Many advances have been made in the last decade. Indeed, since the first micromixers appeared at the beginning of the century, approximately one hundred functional devices based on various concepts have been developed. All of them have advantages and disadvantages, but on the whole, users often find geometries corresponding to their application of interest in the catalog of microfluidic mixers^1–3^. In recent years, the idea of using these devices to produce LNPs (lipid nanoparticles) has emerged^4,5^. LNPs have emerged as the gold standard for nucleic acid delivery^6^. They are complex nanoparticles, 50–100 nm in diameter, composed primarily of cationic ionizable lipids that can segregate from the other lipid components when their charges are neutralized, leading to the formation of amorphous oil droplets in the core of LNPs, as described in a recent study^7,8^. The therapeutic molecule in the LNP depends on the application. It can be DNA, mRNA or siRNA. The functional entities entrapped or adsorbed at the interface include PEG moieties (usually linked to a lipid chain), helper lipids, and cholesterol. Lipid nanoparticles offer many advantages over previous lipid-based nucleic acid delivery systems: high nucleic acid encapsulation efficiency, more potent transfection, improved tissue penetration and low cytotoxicity and immunogenicity. These characteristics make lipid nanoparticles excellent candidates for nucleic acid delivery, as was demonstrated by mRNA-based vaccines against COVID.

LNPs are formed through a self-assembly process. Numerical simulations suggest that the self-assembly process includes three steps: particle assembly into discoidal clusters, aggregation of clusters into larger membrane patches, and vesicle formation^9^. Self-assembly by diffusion would be too slow (it would take days), so hydrodynamic mixing is needed. The use of standard mixers^5^ in large containers is an option. However, these mixers generate size polydispersity along with low encapsulation efficiency. Therefore, postprocessing steps such as filtration, extrusion and centrifugation are required to improve the quality of LNPs produced in this way. In this context, the use of microfluidic mixers is relevant. It has recently been demonstrated that microfluidics allows the production of LNPs of acceptable quality in terms of monodispersity and encapsulation yield in a single step with high yields. In Fujishima et al.^10^ and Shepherd et al.^11^, a staggered herringbone micromixer^12^ was used. One limitation of herringbone micromixers is their low throughput. This limitation can be circumvented by parallelizing the system^11^. However, this option generates complexity, increases the cost and reduces the reliability. Inertial micromixers that operate at significantly higher flow rates and thus higher throughputs offer a solution, but thus far, although some indications can be found in the literature, the conditions under which they should be operated to obtain functional LNPs have not yet been fully elucidated.

In the present paper, we focus on a Dean-based micromixer^13–17^, a member of the class of inertial micromixers. It is well adapted to high throughputs and thus to mass production. We identify, in good agreement with numerical calculations^18^, three domains of flow rates. One of them, called the ‘transition regime’, will play an important role in identifying the optimal flow conditions. To obtain LNPs with optimal characteristics in terms of encapsulation efficiency (EE), size, charge and monodispersity, we show that we must operate at flow rates above the transition regime. Working below or within the transition regime, still in terms of flow rates, leads to degraded performance. In agreement with the literature, we thus outline the physical conditions under which LNPs of acceptable quality can be obtained using a physical criterion that has not been previously proposed. We also discuss the important role of the flow rate ratio between the aqueous phase (containing the nucleic acids) and the organic phase (containing the lipids).

## Materials and methods

### LNP formulation

In our LNP formulation, 1,2-dioleoyl-sn-glycero-3-phosphocholine (DOPC), 1,2-dimyristoyl-rac-glycero-3-methoxypolyethylene glycol-2000 (DMG-PEG2000) and cholesterol (plant derived) were purchased from Avanti Polar Lipids (Alabaster, AL, USA). Dlin-MC3-DNA, henceforth termed MC3, was obtained from SAI Life Science (Hyderabad, India). Citric acid and sodium citrate tribasic dehydrate were acquired from Sigma–Aldrich (France). Phosphate-buffered saline 10X (PBS, pH 7.4) was obtained from Thermo Fisher Scientific (France). The gWiz-GFP plasmid was purchased from Aldevron (North Dakota, USA).

To produce LNPs, lipids were solubilized in ethanol at molar ratios of 50:10:38.5:1.5 for MC3, DOPC, cholesterol and PEG-lipid, respectively. The lipid mixture was mixed with 50 mM citrate buffer at pH 4 containing pDNA using the NxGen microfluidic cartridge (from Precision NanoSystems, Vancouver). The nitrogen to phosphate ratio (N/P) between the ionizable lipid and the pDNA was maintained at 6. The effects of the flow rate (FR; ranging from 0.4 to 20 mL/min), the aqueous to organic ratio (FRR; from 1:1 to 10:1) and the final lipid and pDNA concentrations (from 1.44 to 15 mg/mL and 93–965 µg/mL, respectively, for lipids and pDNA) were evaluated. For all formulations, the initial and final waste volumes were set to 0.45 and 0.05 mL, respectively. After the formulation was processed in the micromixer, ethanol was removed from the product, and the citrate buffer was removed by PBS using Amicon Ultra Centrifugal Filters (EMD Millipore, Billerica, MA). The formulations were finally passed through a 0.22-μm filter and stored at 4 °C until use.

### Measurements of nanoparticle size, polydispersity and zeta potential

The particle size, polydispersity index (PDI) and zeta potential were measured by dynamic light scattering using a Malvern Zetasizer NanoZS (Worcestershire, UK). LNPs were diluted 100 times in PBS and added to a µ-cuvette. The dispersant (PBS) refractive index (RI) and vicosity values were 1335 and 1.02 cP, respectively, whereas the material RI was 1.45.

### Quantification of nucleic acid loading

The pDNA encapsulation efficiency was determined using the PicoGreen DNA assay (Life Technologies, Burlington, ON). Briefly, 100 µL of the diluted fluorescent dye was added to 100 µL of diluted LNPs in the presence or absence of 1% (w/v) Triton-X100 in TE buffer and incubated in the absence of light for 5 min. Nucleic acids were quantified by measuring fluorescence (ex/em = 480 nm/520 nm) using a fluorimeter (Varioskan Lux Microplate reader, Thermo Fisher). A linear calibration curve up to 1000 ng/mL was performed using the standard DNA sample provided in the kit.

The encapsulation ratio was calculated by the following formula:

$${\text{EE }}\left( {\text{\% }} \right){ } = \left( {1 - \frac{{{\text{pDNA concentration in absence of triton}} }}{{\text{pDNA concentration in presence of triton }}}} \right) \times 100$$

### CryoTEM analysis

The morphology of the LNPs was observed by cryo-electron microscopy. A total of 4.1 µL of concentrated LNPs was deposited on Quantifoil R2/2 copper 300 mesh grids (Quantifoil Instruments GmbH, Germany) after 90 s of glow discharge on an ELMO ionizer (Cordouan, France). Grids were blotted and frozen using a Vitrobot MARK IV (Thermo Fisher Scientific, USA) and transferred onto a TEM tecnai-G20 (ThermoFisher Scientific, USA) operated at 200 kV using a 910 cryo-holder (Gatan, Inc., USA) for observation. Images were recorded at 4 µm defocus and in low-dose mode (electron doses between 10 and 15 e−/Å2) using an ssCCD Ultrascan 4000 (Gatan, Inc., USA). The pixel size of the recorded images was estimated to be 0.221 nm after TEM calibration using a cross line grid (EMS, USA) with pitch spacing of 500 nm and 2000 lines/mm.

### Microfluidic device, channel geometry and flow conditions

**Figure 1:**
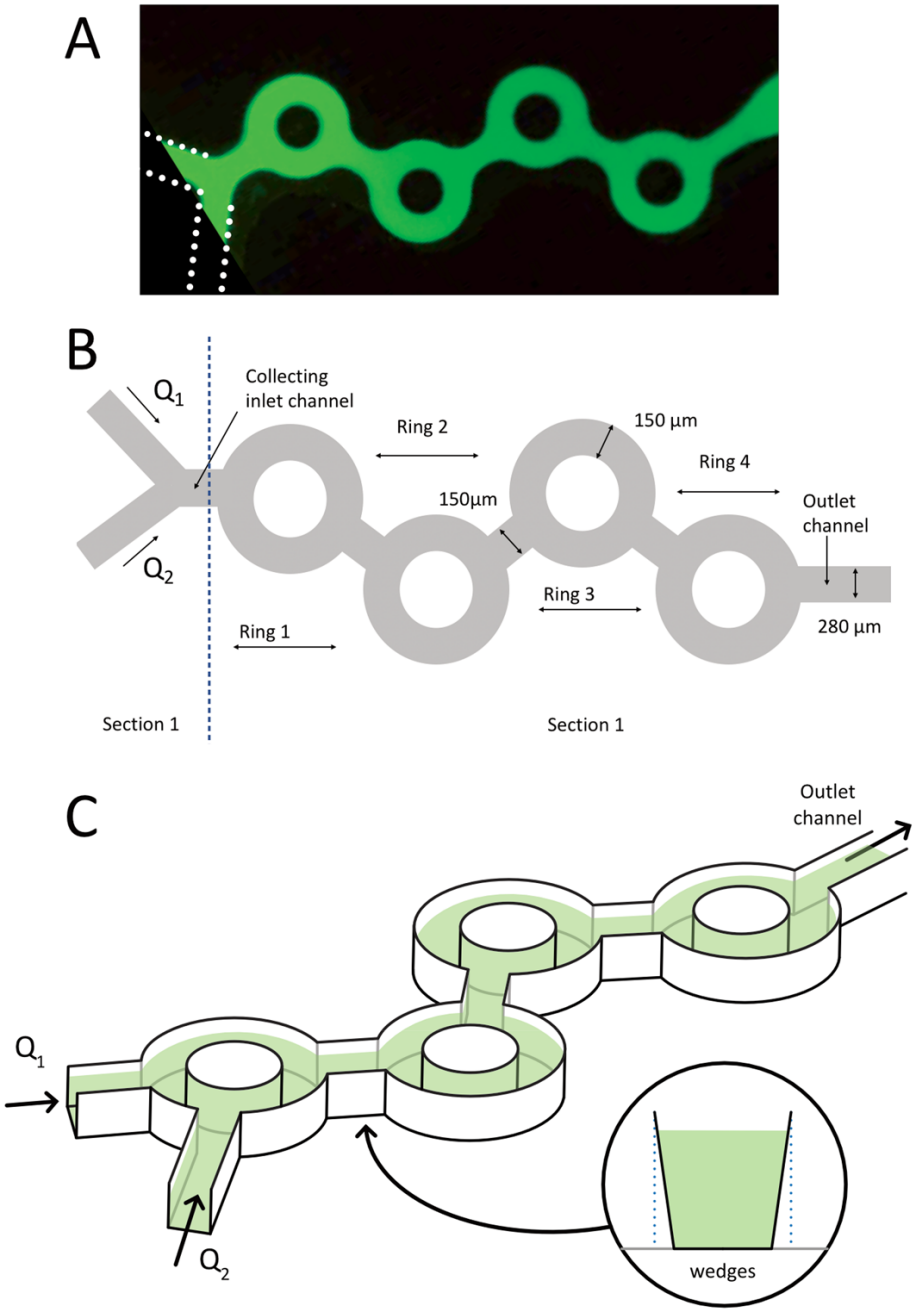
(**A**) Image of the microdevice filled with a fluorescein solution. (**B**) Sketch of the microdevice showing Sect. 1, the entry region, and Sect. 2, including the four rings and the junctions between them. In the fluid model experiments, Q_1_ is water, and Q_2_ is ethanol with 0.1% w/w fluorescein. In the LNP experiments, Q_1_ represents the aqueous phase containing the nucleic acid diluted in an acidic buffer, and Q_2_ represents the ethanol phase containing the lipids. (**C**) Three-dimensional view of the device, showing the wedges associated with the molding technique of microfabrication. The dimensions of the channels were measured by optical profilometry (see Fig. 2).

The microfluidic device used to produce LNPs is shown in Fig. 1.

**Figure 2:**
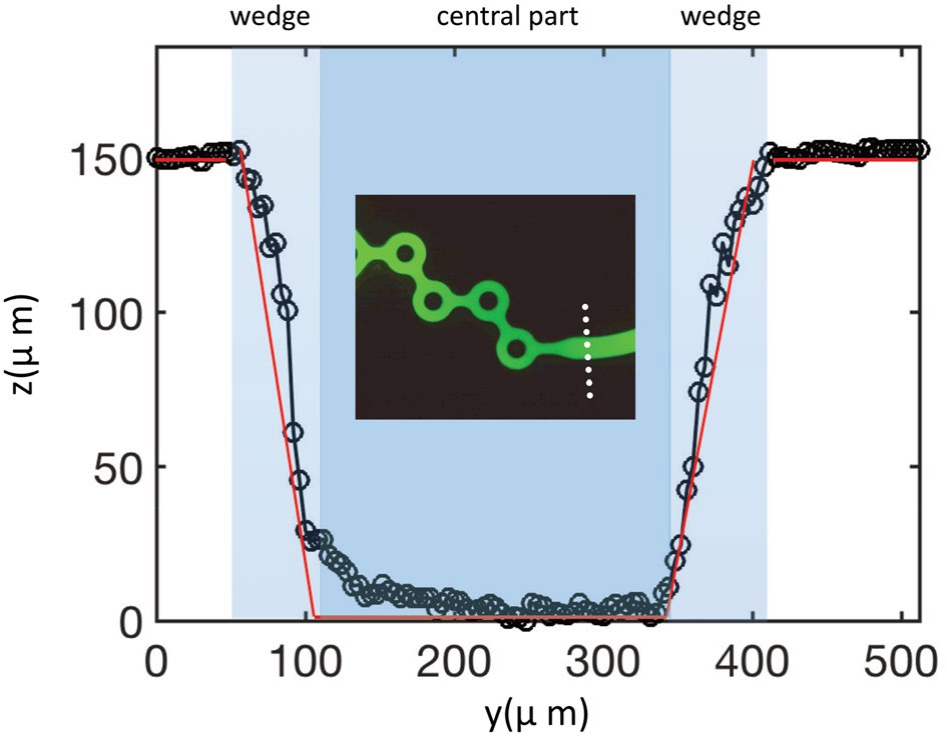
Cross-sectional profile of the outlet channel (see the dashed line in the insert), with *y* representing the horizontal dimension (i.e., in the plane of the device) and *z* the height. The function *z*(*y*) thus defines the channel profile, with the channel bottom taken as a reference (*z* = *0*). Two sets of data are shown: intensity measurements (circles), assuming proportionality between the fluorescence intensity and *z*(*y*), and Veeco optical profilometry measurements (red lines).

The system included a series of four tori connected by straight channels. The device was microfabricated with plastic molding technology (Ignite® from Precision NanoSystems Inc. Ltd., Vancouver, BC, Canada). Figure 2 shows the cross-section of the outlet channel located downstream of the four rings, as shown in the inset (see the white dashed line).

The profile in Fig. 2 was obtained by filling the channels with a solution of alcohol and fluorescein, imaging them with a fluorescence microscope, collecting the intensity field with a camera and analyzing the images with a computer. The larger the height of the channel is, the stronger the collected intensity. Working with low fluorescein concentrations (0/1% w/w) to avoid bleaching, we assumed proportionality between the fluorescence intensity collected by the camera and the channel height. We checked that the profiles obtained in this way were in full agreement with optical profilometry (see the red line of Fig. 2, obtained with the optical profilometer Veeco). Due to the microfabrication technology used (injection molding), the channel walls were not vertical but slightly inclined. From Fig. 2, angles of approximately 80° were obtained, forming what we call “wedges” by opposition with the central part of the channel for which the height is constant. In this context, we define the channel width *w* as the distance between the two inclined walls at the mid-plane level. In our mixing analysis, we will neglect these wedges and focus on the central parts, which transport most of the material we want to produce (in the wedge, because of the confinement, the fluid, subjected to the same pressure gradient as in the central part, is essentially stagnant, and we may assume that it does not contribute to the production process). The surface roughness estimated from optical profilometry was 3 ± 1 µm. This is typical of injection technology without surface postprocessing. As the flow is driven well below turbulence onset (see below), this roughness does not play any dynamical role.

The channels have the same depth (155 µm -measured by optical profilometry -) and different widths. In Fig. 1, the tori are 150 µm wide, the connecting channels 150 µm, and the inlet and outlet channels 280 µm wide (as shown in Fig. 2). The device has two inlets. The entry channels form a Y that is connected to the first torus.

The total flow rate is defined by *Q* = *Q*_*1*_ + *Q*_*2*_, where *Q*_*1*_ and *Q*_*2*_ are the flow rates of the fluids injected at the two inlets (see Fig. 1). In our system, two types of experiments were conducted:

- *Model fluid experiments*: In this case, Q_1_ corresponds to DI water and Q_2_ to the fluorescein ethanol solution at a 0.1% mass concentration.
- *LNP experiments*: In this case, Q_1_ represents the aqueous phase containing the nucleic acid in an acidic buffer, and Q_2_ represents the ethanol phase with the four solubilized lipids.

We also define the FRR (flow-rate ratio) by the relation $QR = \frac{{Q_{1} }}{{Q_{2} }}$. Most of the work focused on FRR = 3, i.e., a water flow rate of three times the ethanol solution flow rate. The total flow rate Q was varied from 0.2 to 20 ml/min. We define a characteristic speed U as the total flow rate Q divided by the inlet channel cross section, i.e., just before ring 1 (see Fig. 1). The Reynolds and Dean numbers are defined by the following expressions:

$${\text{Re}} = \frac{Uh}{\nu }\,\,{\text{and}}\,\,De = {\text{Re}} \sqrt{\frac{h}{2R}}$$

where *h* is the channel height, ν is the kinematic viscosity of water, and R is the curvature radius of the tori. In the range of flow rates we tested, the Reynolds numbers vary from 10 to 1100, while the Dean numbers range between 4 and 400. We were thus well below the turbulence development regimes, which might have required fine control of the wall roughness. From a dimensional viewpoint, our system depends on these two numbers, along with aspect ratios and a dimensionless number characterizing the diffusion process. The parameter space is large, and in this paper, we will plot the observables as a function of the flow rates (which, from a practical viewpoint, are useful) without attempting to generalize the conclusions by using dimensionless numbers.

### Method of characterization of the mixing process

For the fluid model experiments, characterization of the mixing process was performed by using visualization techniques. Fluorescein was injected at one entry, and the fluid injected at the other entry contained no dye. The system was observed with a fluorescence microscope equipped with a source at 480 nm and a camera filter at 520 nm, i.e., around the emission peak.

As classically done in the literature^19^, the study of the fluorescence intensity field provides information on mixing. Similarly, we define a mixing index *H* by the formula:

$$H = 1 - \sqrt {\frac{{\mathop \smallint \nolimits_{0}^{w} \left( {I - I_{mean} } \right)^{2} dy}}{{wI_{mean}^{2} }}}$$

in which *y* is the coordinate transverse to the flow, *w* is the channel width, and $I_{mean}$ is the average intensity across the channel width. By definition, when *H* is close to unity, mixing is high. In the opposite case (small *H*), mixing is low.

## Results

### Results on the flow patterns and the mixing characteristics for FRR = 3 in the fluid model experiments

**Figure 3:**
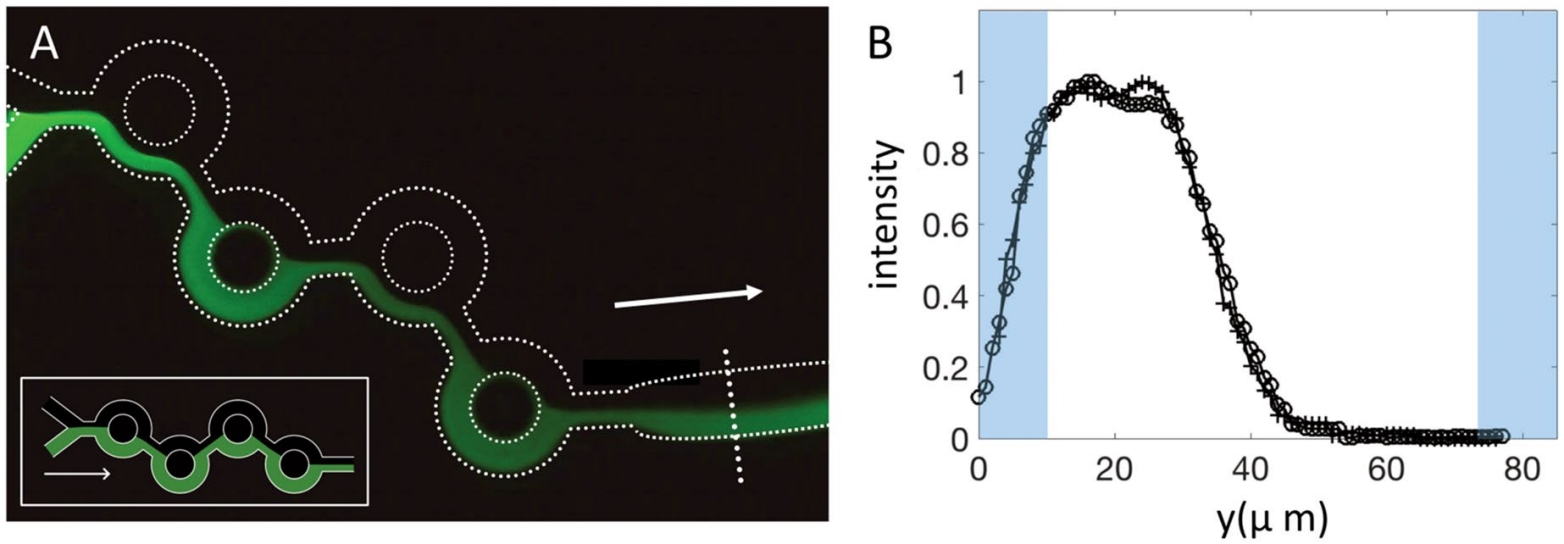
(**A**) Fluorescence field of the micromixer for a total flow rate Q equal to 0.2 ml/min. Insert: Theoretical tracer distribution if the dye followed the streamlines without diffusion, for which we assumed no recirculation. (**B**) Intensity profiles in the outlet section of the system for Q = 0.2 (open circle) and 0.3 (crosses) ml/min. The dashed line shows the center of the diffusive layer, and the gray areas show the wedges discussed in the Materials section. The measurements were performed in the outlet channel, i.e., downstream of the fourth ring (see the dashed line). Gray zones are the channel wedges.

We first considered low flow rates, keeping the flow-rate ratio FRR equal to 3. The choice of this value was motivated by the fact, as shown later, that it enabled the formation of LNPs with the appropriate sizes for DNA or RNA delivery (approximately 100 nm), a narrow size distribution and an excellent encapsulation efficiency. A typical image of the fluorescence field at low flow rates is shown in Fig. 3A.

In Fig. 3, the flow rate Q_2_ of the ethanol solution is 0.05 ml/min, while water is injected at a flow rate Q_1_ equal to 0.15 ml/min, so that the total flow rate Q = Q_1_ + Q_2_ is 0.2 ml/min. As stated above, the flow rate ratio FRR is equal to 3. Figure 3 indicates that in the collecting entry channel, the two fluids travel side by side. The intensity profile in Fig. 3B shows the existence of a diffuse layer. The measured thickness of the diffuse layer was on the order of 60 µm, which was larger but of the same order of magnitude as the estimate $l \sim 6\sqrt {DT }$ of diffuse layers based on the error function (where D is the fluorescein diffusion constant in ethanol (2 10^–10^ m^2^/s) and T is the travel time (30 ms for 0.2 ml/min)). The formula leads to a thickness *l* on the order of 30 µm. We suggest that the factor of 2 is due to the action of weak recirculations, present in the system even at low flow rates, localized at the entrance or developing along the rings. However small these recirculations may be, they may significantly enhance diffusive transport.

Figure 3A thus shows that ethanol and water flow side-by-side. In such a regime, the following expressions hold^19,20^.

$$w_{e} = \frac{w}{1 + \alpha },w_{w} = \frac{\alpha w}{{1 + \alpha }} \,\,{\text{with}}\,\, \alpha = \frac{{\mu_{w} Q_{1} }}{{\mu_{e} Q_{2} }}$$

in which µ_e_ and µ_w_ are the viscosities of ethanol and water, respectively. The formula says that the fluorescein ethanol mixture occupies 30% of the channel width. This compares well with Fig. 2, in which the dashed line marking the center of the diffuse layer is located at 35% of the channel width. The inset in Fig. 3A shows the flow pattern we would expect if there was no swirling motion and if diffusion was neglected.

**Figure 4:**
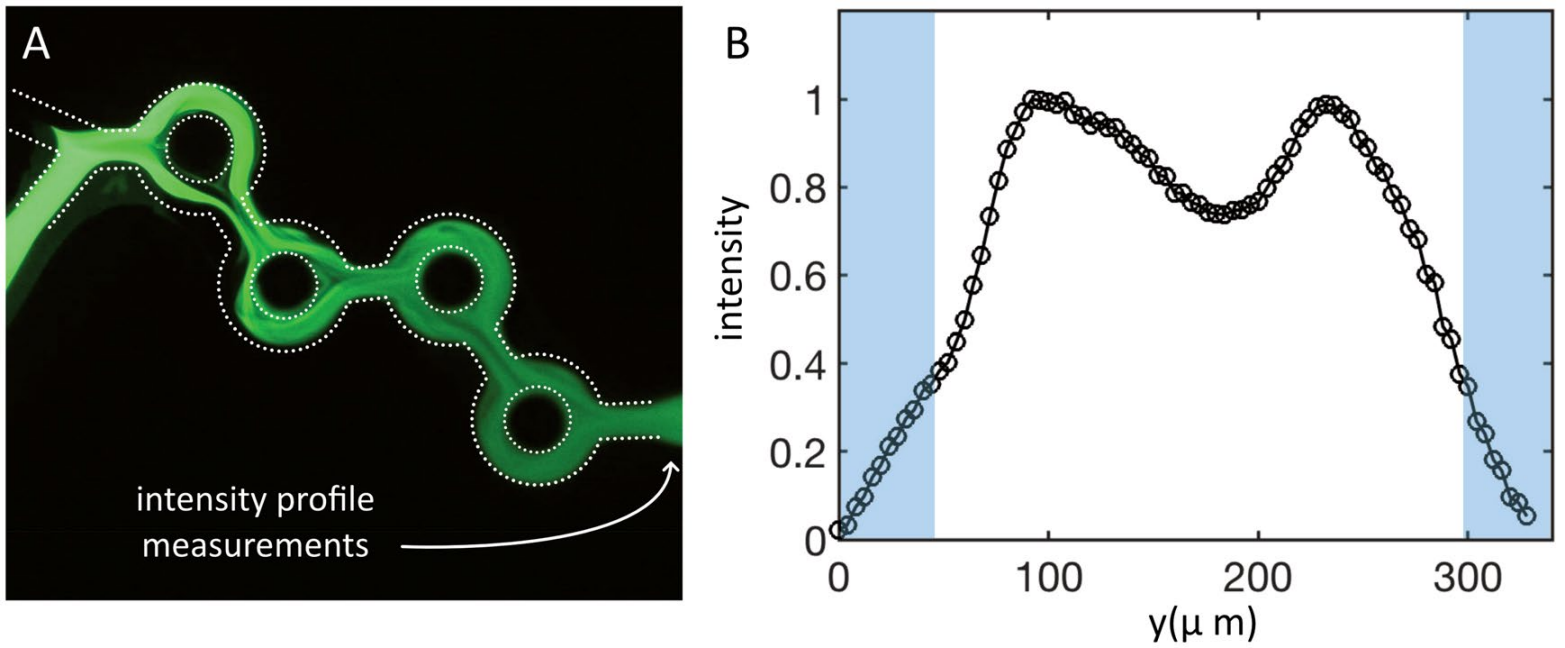
Fluorescence field of fluorescein transported in the device by ethanol for Q_1_ = 1.5 ml/min (water) and Q_2_ = 0.5 ml/min (ethanol): (**A**) Instantaneous image of the fluorescence field. (**B**) Time-averaged (over one minute) intensity profile measured after the fourth ring (see the arrow), i.e., in the outlet channel. Gray zones are the channel wedges.

As the total flow rate Q was increased in the range of 0.7–4 ml/min, still with FRR = 3, the time-averaged concentration profiles adopted complicated shapes that varied substantially from one flow rate to another, even though the differences between two successive values were as small as 10%. Thus, there is high variability in this region. We call this domain the ‘transition domain’ and the corresponding regimes ‘transition regimes’. Figure 4A shows a typical concentration field and Fig. 4B a typical concentration profile obtained in this regime, measured again, in the outlet microchannel (see the arrow).

In this case, the tracer invaded the channel but not completely: the concentration profile, which is averaged in time for one minute, shows a pronounced dip around the centerline. The mixing was therefore not complete because some regions were well mixed and others are less mixed. An important observation is that in the transition regime, fluorescein invaded the channel before entering the first torus. This suggests that a transport mechanism developed at the junction where the two fluids met. As indicated in Fig. 3A, the dye concentration tended to homogenize as we moved downstream, but heterogeneities still remained in the outlet channel, as shown in Fig. 3B.

**Figure 5:**
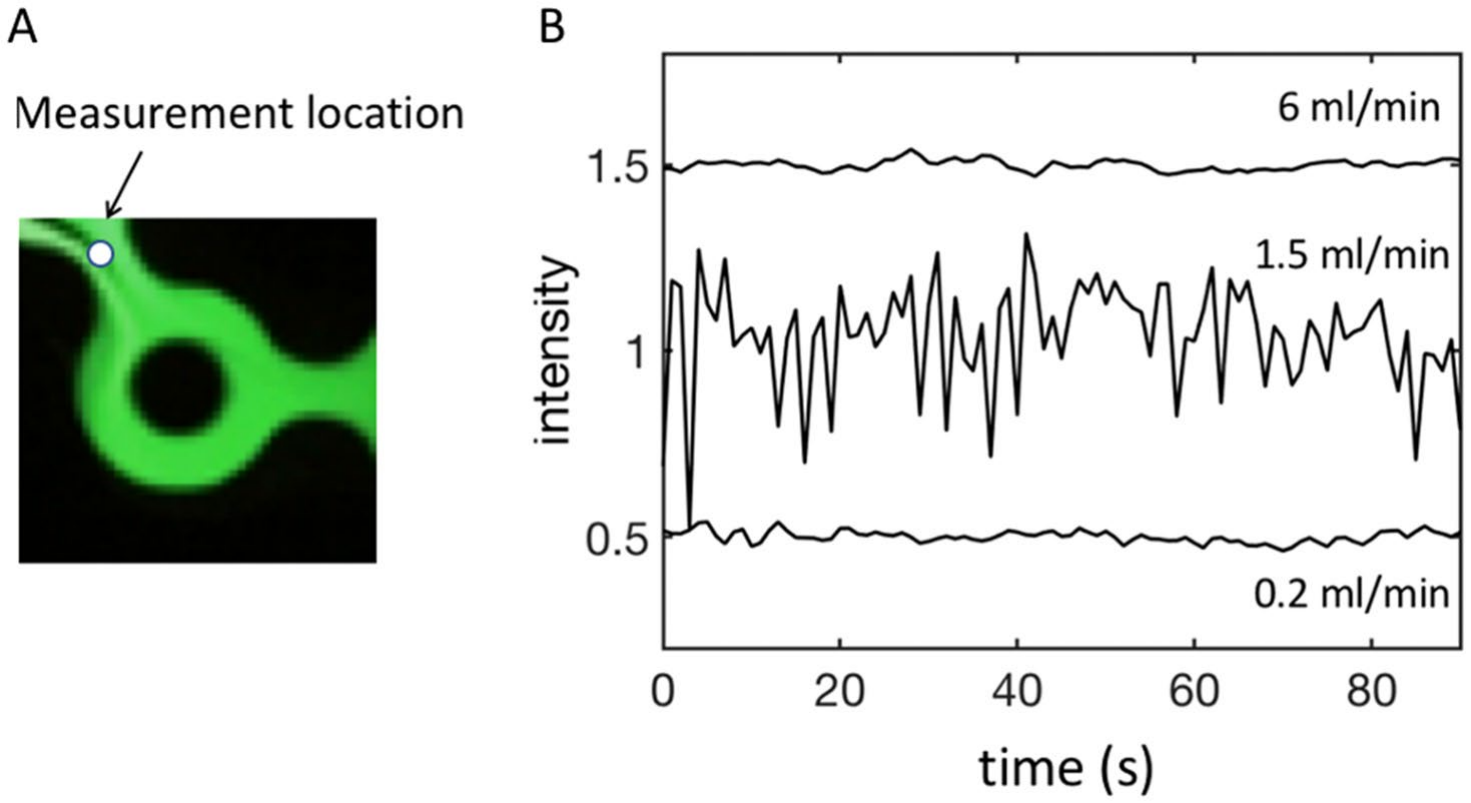
(**A**) Instantaneous fluorescence image taken just before Ring 2 for Q = 1.5 ml/min, always keeping the FRR equal to 3. (**B**) Instantaneous intensity measured at the point indicated by the arrow on the left figure. The positions of the sharp interfaces varied over time, giving rise to oscillations in the local fluorescence intensity appearing on the intensity-time plot. The Reynolds numbers corresponding to the flow rates shown on the plot are, from the lowest to the highest flow rates, 11, 80 and 330.

An interesting feature we observed in the 1–4 mL/min range was the presence of time-dependent phenomena, as shown in Fig. 5.

**Figure 6:**
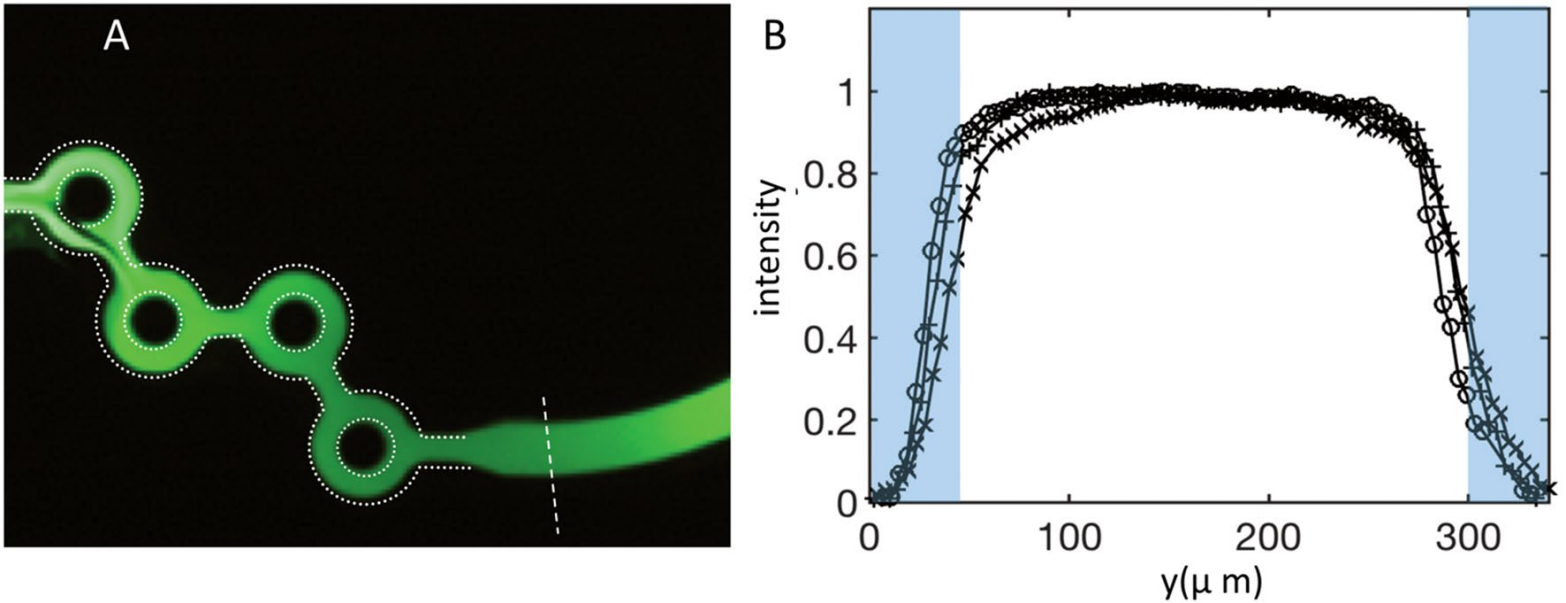
(**A**) Fluorescence field developed by the micromixer for Q = 20 ml/min; (**B**) Three time-averaged concentration profiles obtained for Q equal to 6 (circles), 14 (crosses) and 20 (plus) ml/min. Gray zones are the channel wedges.

To obtain Fig. 5, we measured the instantaneous intensity of fluorescence at a fixed point for different flow rates. The position of the measurement point is indicated with a white dot in Fig. 5A. Figure 5B shows that at 0.2 ml/min, there was no significant oscillation of the local intensity. At 1.5 ml/min, local oscillations induced by fluorescein/water interface displacements were visible. Their amplitude vanished at higher flow rates (see the curve at 6 ml/min). These measurements provide evidence for the presence of time-dependent phenomena in the system. They developed in a narrow range of flow rates, located roughly between 1 and 4 ml/min. We hypothesize that at larger flow rates, the flow was still unsteady, but the coupling between the hydrodynamic oscillations, which tend to striate the fluorescence field, as currently observed in chaotic mixing^19^, and molecular diffusion, which efficiently smooths it out, eventually produced a homogeneous concentration field. The limiting case of complete mixing is well illustrated in Fig. 6A.

The figure shows that the tracer, apart from a small region around the first ring, is homogeneously spread throughout the device. The corresponding concentration profiles are shown in Fig. 6B for three flow rates, 6, 14 and 20 ml/min, still keeping FRR = 3. These profiles, again measured at the level of the collecting channel, close to the outlet (see the dashed line in Fig. 6A), collapse onto each other.

**Figure 7:**
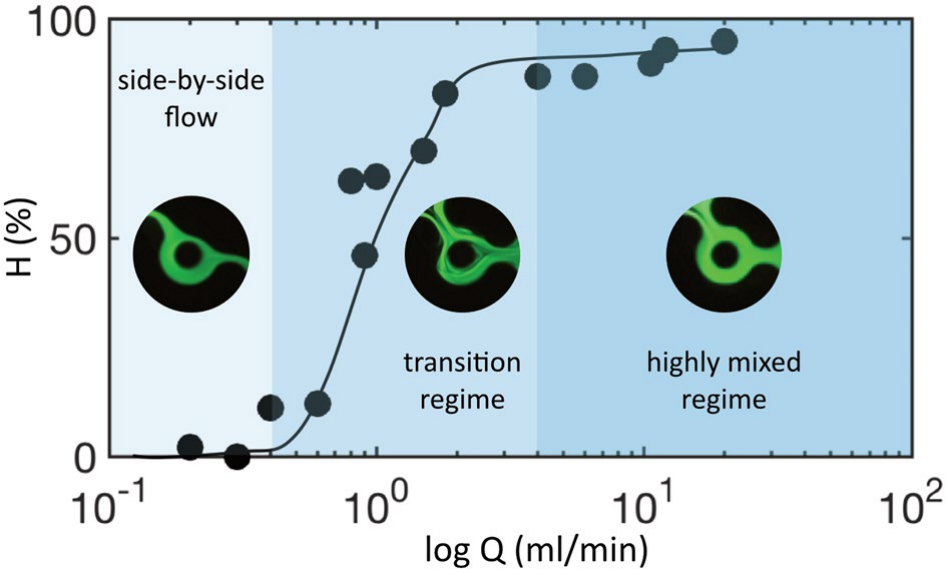
Evolution of the mixing index H as a function of the total flow rate Q for an FRR equal to 3, with typical instantaneous images of fluorescein. Each point results from an average of one minute. The full line has been plotted to guide the eyes.

Figure 7 represents the evolution of the homogeneity factor H, defined previously, measured in the outlet channel for a range of flow rates embracing the three selected regimes, keeping again the flow-rate ratio FRR = Q_1_/Q_2_ equal to 3.

The data are plotted on a semilogarithmic scale, and the homogeneity factor H was obtained by excluding the wedges, considering, as said above, that they do not significantly contribute to the production process. The three regimes we identified from these measurements are the following:

- Q < 0.4 ml/min: Poor mixing, associated with side-by-side flows. The homogeneity factor H is small, approximately a few percent. The corresponding upper Reynolds number of this regime is 22.
- 0.4 < Q < 4 ml/min: Transition regime, associated with moderate mixing, along with complex concentration profiles and time-dependent phenomena. The homogeneity factor H lies between 20 and 80%. In terms of Reynolds numbers, the domain ranges between 22 and 220. The variation in H from one measurement to the other, even though they are averaged over long times compared to the characteristic time of the oscillation, is typical of systems in which flow instabilities develop. This dispersion is consistent with the remarks made above concerning the variability of the concentration profiles with the flow conditions.
- Q > 4 ml/min: Highly mixed regime with homogeneity factors above 80% and flat, reproducible concentration profiles. The Reynolds number range lies between 220 and the maximum value reached in the experiment, i.e., 1100.

For discussing Fig. 7, it is interesting to split the system into two parts, as sketched in Fig. 1B:

- *Sect 1*: The entry region, including the V junction and the channel of connection to the first ring.
- *Sect 2:* The rest of the system, including the rings, the junctions between two successive rings and the outlet microchannel.

#### Section 1

Our observations can be compared to those of Minakov^18^, in which two miscible fluids were injected at a T-junction into a straight channel. The geometry was thus similar to ours, except that we used a Y-junction rather than a T-junction. The results of this work were qualitatively confirmed experimentally^21^. According to these references, above a certain threshold estimated to be Re ≈ 20, symmetric, S-shaped Dean-type vortices develop in the junction. This threshold is close to ours (estimated to be 22). Above this regime, an asymmetric steady pattern develops, up to Re ≈ 150. The development of oscillatory instabilities appears at 240, inducing a periodic displacement of a saddle point, which, according to the Poincaré-Melnikov scenario, gives rise to chaotic mixing. In effect, Minakov’s work shows efficient mixing just after the onset of time-dependent flows^18^. This range of Reynolds numbers is consistent with the regime we called the ‘transition regime’, in which the side-flow regime does not hold, mixing is moderate, heterogeneities are present, and oscillations develop. In our case, the transition regime extends from a Reynolds number equal to 22–220, which is not as broad as Minakov’s results but consistent with it. We may thus suggest that our ‘transition regime’ embraces shaped eddies, asymmetric eddies and oscillating eddies. Oscillating eddies are known to be extremely efficient from the perspective of mixing^19^. This may explain why, just above the onset of oscillations, we reach what we call ‘highly mixed regimes’. Therefore, comparison with the work of Minakov provides clues to understand, on a semiquantitative basis, the behavior of our micromixer.

#### Section 2

There is evidence that the four rings improve dye homogenization. This is illustrated in Fig. 5, which shows that inhomogeneities at the entry of the device tend to be smoothed out as we move downstream. In the rings, two parts can be singled out: one is the ring itself (Part 1), and the other (Part 2) is the collecting channel, i.e., the region where the two fluids meet again. Part 1 presumably plays a minor role in mixing because, compared to the junctions (i.e., Part 2 or the V entry), the curvature is smaller, and therefore the Dean vortices that may develop are weaker. On the other hand, Part 2 may be regarded as a Y-junction, playing a role similar to the entry, thus improving mixing. In fact, the experimental observations we made suggest that mixing events first start at the entry, i.e., in Sect. 1, and repeat in Part 2 of the rings. Therefore, we can suggest that Part 2 complements the work done by Part 1 regarding mixing but does not initiate it.

### The correlation between mixing and LNP characteristics for a flow-rate ratio FRR equal to 3

The question we now address is the relationship between the mixing characteristics as previously analyzed and the LNP properties obtained with the same micromixer. To perform these investigations, an LNP-pDNA model was used. LNPs were fabricated using the ready-to-use and commercially available gWiz-GFP plasmid^22,23^ and four different lipids with optimal mole ratios of ionizable-lipid/DSPC/cholesterol/PEG-lipid of 50/10/38.5/1.5 and an N/P ratio of 6 for the amine group of ionizable lipid to the phosphate groups of pDNA as described in the literature^24,25^. The MC3 ionizable lipid (DLin-MC3-DMA) was chosen because it is the most clinically advanced oligonucleotide delivery system^26,27^ and is also commercially available. The molar ratio between all components was kept constant and chosen according to the literature.

We thus investigated the effect of the total flow rate Q on the LNP size, size variability (PDI—polydispersity Index), ζ potential, and encapsulation efficiency (EE). This work represents the ‘LNP experiments’ previously defined. These key characteristics are usually measured from the early development stages to control the process and ensure the effectiveness and safety of the final product. According to the literature, ideally, LNP sizes should be approximately 100 nm or less to ensure optimal biodistribution and efficient drug delivery^28^; PDI should be lower than 0.2 to certify the product homogeneity; and zeta potentials should be slightly negative and encapsulation efficiency as high as possible to ensure high process yield and nucleic acid protection.

**Figure 8:**
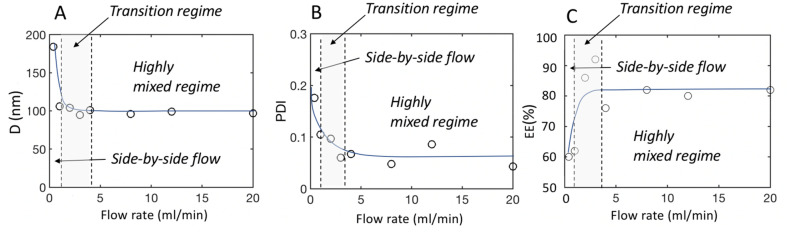
Evolution of various quantities with the flow rate, between 0.4 and 20 mL/min (**A**) LNP Sizes; (**B**) PDI (polydispersity index of the LNPs); (**C**) Encapsulation efficiency.

To analyze the correlation of these parameters with the flow conditions, five flow rates Q ranging from 0.4 ml/min to 20 ml/min were studied. In these experiments, in the same manner described above, the ratio between aqueous and solvent streams was maintained equal to 3, and the total lipid concentration (1.44 mg/l) and the chemical composition were kept constant. The data are shown in Fig. 8.

Figure 8A shows that larger particles were obtained at low Q (< 1 ml/min). After a transition zone in which the sizes seemed to decrease, the particle sizes reached a plateau at approximately 100 nm at higher flow rates, i.e., between 4 and 20 ml/min. In the meantime, the nanoparticle dispersity (PDI) decreased with increasing flow rate, levelling off for Q > 4 ml/min at 7% (see Fig. 8B). In parallel, the EE was approximately 60% below 2 ml/mn, while particles formed at higher Q, above 4 ml/min, had an EE of approximately 80% (see Fig. 8C). These results were consistent with the results of the mixing study we performed. We have plotted, on the same graph, the boundaries of the three zones we singled out in Fig. 7 i.e., the poorly mixed, transition and highly mixed regions. Notably, the characteristics of the LNP structure correlate well with the mixing characteristics of the device. In addition, the ζ potential, indicating the effective surface charge^29^, was also measured. Regardless of the flow rate used, all LNPs exhibited a zeta potential of ζ = − 13 mV + /− 4 mV at pH 7.4 and were thus slightly negative. The measurement was consistent with Ref^25^ and suggested a loss of the positive charge of the ionizable lipid.

We may conclude, from a practical perspective, that when the mixing is not satisfactory (i.e., with a homogeneity index H below 80%), the properties of the structures that are created are not optimal, whereas they reach an optimum when mixing is high (H larger than 80%) and the intensity profiles are homogeneous across the channel. We suggest that working above the transition zone, more specifically, substantially above the conditions for which oscillatory flows develop, leads to optimal LNPs.

### Evolution of LNP characteristics with the flow-rate ratio (FRR)

**Figure 9:**
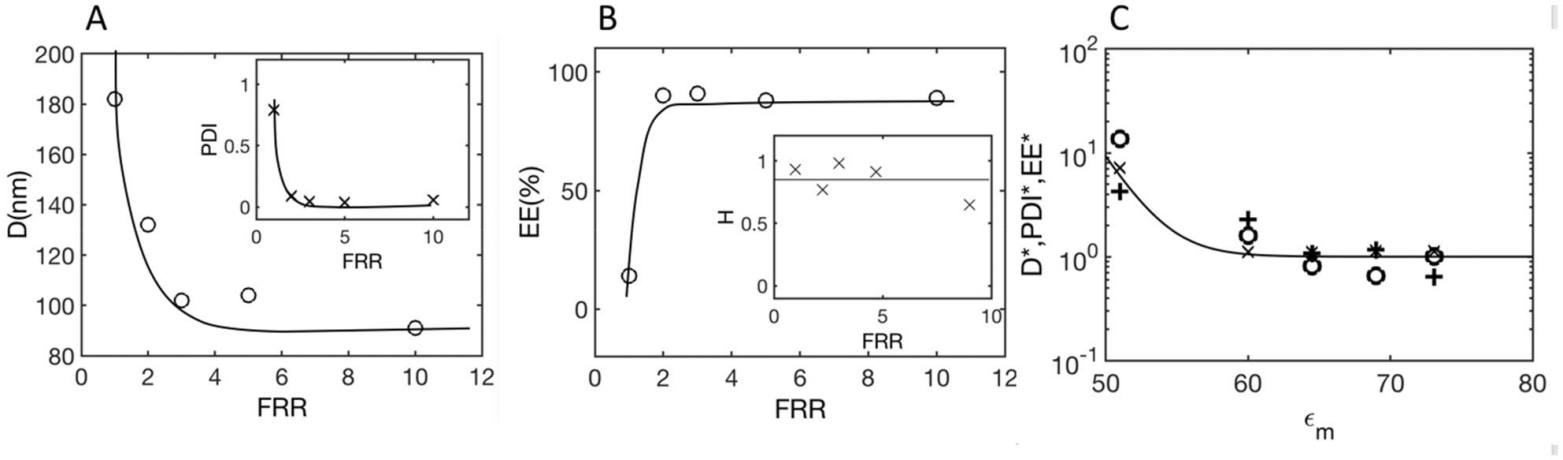
Graphs obtained for a total flow rate of Q = 4 ml/min. (**A**) LNP diameter D as a function of FRR (i.e., water over ethanol solution flow-rate ratio). Inset: PDI as a function of FRR. (**B**) encapsulation efficiency EE as a function of FRR. Inset: Homogeneity factor H as a function of FRR. (**C**): Normalized quantities plotted as a function of $\varepsilon_{m} = \frac{{FRR\varepsilon_{w} + \varepsilon_{e} }}{FR + 1}$; $D^{*} = 1 + 4\frac{{D - D_{\infty } }}{{D_{\infty } }}\left( + \right);$ $PDI^{*} = PDI/PDI_{\infty }$(o) and $EE^{*} = E_{\infty } /EE$ (x).

It is important, as described in Roces et al.^5^, to address the role of the flow-rate ratio FRR. Figure 9 shows the results obtained for the LNP diameter D, the PDI, and the encapsulation efficiency EE for a total flow rate of 4 ml/min and different values of FRR, varying from 1 to 10.

For an FRR smaller than 2, the LNP characteristics were not ‘optimal’ (in the sense defined above): diameters above 100 nm (Fig. 9A), low EE (inset of Fig. 9B) and high PDI (Fig. 9C) were observed. At a larger FRR, we recovered the optimal characteristics, i.e., diameters close to 100 nm, large EE and small PDI (on the order of 6%). It is important to note, as shown by the insert in Fig. 9B, that in all cases, i.e., at all FRRs, mixing was high: the homogeneity factor H was on average approximately 80%. We may infer that the origin of the structural pathologies of the LNP, for FRR < 2, was not due to insufficient mixing.

**Figure 10:**
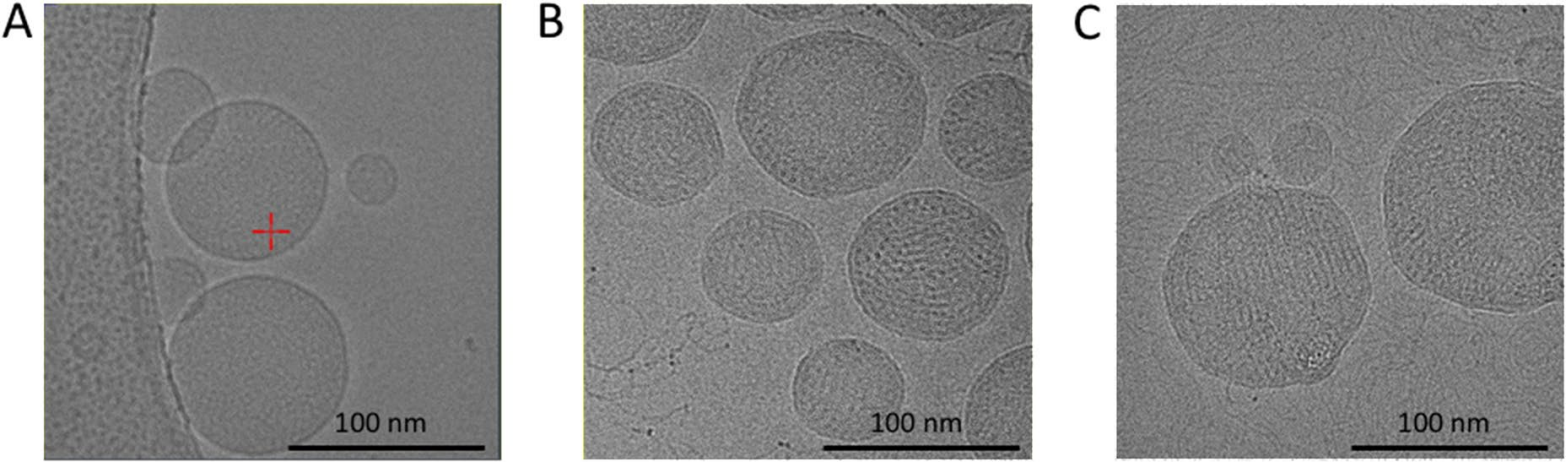
Cryo-TEM images of LNPs composed of MC3, DOPC, PEG-lipid and cholesterol with a molar composition of 50/10/1.5/38.5, respectively, obtained at different flow rates and with an FRR of 3. (**A**) LNPs prepared in the absence of nucleic acid at 4 mL/min. (**B**) pDNA-LNP systems prepared with p-DNA at 4 mL/min, showing more textured surfaces. (**C**) pDNA-LNP systems prepared at 0.4 mL/min. Sizes are larger and distributions broader (see the presence of small and large LNPs).

Figure 9C allows us to propose an explanation. When ethanol (relative dielectric constant ε_e_ = 25) and water (relative dielectric constant ε_w_ = 78) are mixed, the effective (relative) dielectric constant of the mixture is equal to $\varepsilon_{m} = \frac{{FRR\varepsilon_{w} + \varepsilon_{e} }}{FR + 1}$. Figure 10C shows the evolution of the diameter D, PDI and encapsulation efficiency EE in normalized forms as a function of $\varepsilon_{m}$. Specifically, we represented the following quantities: $D^{*} = 1 + 4\frac{{D - D_{\infty } }}{{D_{\infty } }},$ $PDI^{*} = PDI/PDI_{\infty }$, and $EE^{*} = E_{\infty } /EE$, where ‘$\infty^{\prime }$ is the value obtained at FRR > 4 mL/min. At high FRR, $\varepsilon_{m}$ is close to that of water, and the LNPs have optimal properties, while at small FRR, $\varepsilon_{m}$ is substantially smaller, the energy landscape ‘seen’ by the LNP constituents is changed, and we may hypothesize that this affects the self-assembling process and thereby LNP morphologies in a detrimental manner. This type of situation occurs when important quantities of ethanol are used in the formulation^24,30–32^. From Fig. 9C, we estimate that the crossover between the nonoptimal and optimal cases is located around an effective relative dielectric constant $\varepsilon_{m}$ close to 60, still for Q = 4 ml/min, which corresponds to a flow-rate ratio (FRR) on the order of 2.

From this remark, we may suggest that, optimally, the FRR must be simultaneously large enough to maintain the polarity of the solution close to that of water and small enough to avoid reaching high dilutions, which would decrease the yield of the process.

### Morphology of LNPs in the highly mixed regime with FRR = 3

Here, we analyze the morphology of the nanoparticles produced in the highly mixed regimes at 4 mL/mn and FRR = 3. We used cryo-TEM, considering, for the sake of comparison, LNPs without pDNA (“empty” LNPs) and with nucleic acids (pDNA LNPs). We also compared the results with those of poorly mixed regimes, as shown in Fig. 10.

In the well-mixed regimes, the size (close to 100 nm) observed by cryo-TEM was consistent with the DLS measurements. We found that empty LNPs had smooth spherical shapes, whereas pDNA LNPs showed a corrugated interface (Fig. 10B). It is probable that the action of encapsulated DNA strands on the lipid layer causes this phenomenon. In any case, we found that the electron-dense core structure of Fig. 10 was consistent with the functional acid-based LNPs reported in the literature^5,8,24,33^. We may thus conclude that in highly mixed conditions and with FRR = 3, the structures we found are ‘optimal’ in the sense that they are consistent with the functional LNPs imaged in the literature. When LNPs are prepared under low mixing conditions (0.4 mL/min, “side by side regime”), the same types of structures are observed, but populations appear to adopt larger sizes, with broader distributions, consistent with Fig. 8A.

## Discussion

The results presented above indicate that acceptable LNP quality (low size, low PDI, weakly negative ζ potentials and high EE) is obtained when two conditions are met: a homogeneous mixture of the two phases used for formulating the LNP, with a homogeneity factor larger than 80%, and a phase distribution such that the mean relative dielectric constant of the mixture is larger than 60. This is the range of conditions we may recommend for preparing functional LNPs. Our results suggest that, independent of the mixer geometry, if one of the two conditions is not satisfied, LNP sizes, size homogeneity and encapsulation efficiency will differ substantially from optimal values.

It is interesting to ask why, at low flow rates, anomalous LNPs. Possessing significantly larger sizes and smaller encapsulation efficiencies than the optimal structures are obtained. The fluorescence profile of Fig. 3 shows the presence of a diffuse layer, 60 μm thick, that separates the two fluids. In the center of this layer, the water/ethanol ratio is approximately 1:1. We may infer that in this region, the optimal self-assembly conditions are not met. On the other hand, lipids and pDNA are more massive than water and ethanol and therefore diffuse more slowly. As reagents travel downstream, there will be periods of time in which lipids experience this suboptimal environment and thereby precipitate. This reasoning may explain the anomalous sizes and low performances observed in the poor mixing regimes.

Notably, at the highest flow rates we imposed, the LNPs maintained their integrity, indicating that they did not break up. This can be understood by noting that, despite the high speed (i.e., cm/s), on the scale of the LNP, i.e., 100 nm, the shear stresses are low. Should we increase the system size, there would be no difficulty in raising the flow rate and thus the throughput, provided the shear stress ‘seen’ by the LNP remains at the same level as in our experiments, and the flow conditions remain below the turbulence onset.

Finally, by using the understanding we gained in this work, we may attempt to define optimal conditions for producing LNPs for the type of mixer we used. First, the flow rate must be large enough to achieve high mixing. In our case, a minimum flow rate of 4 ml/min can be proposed. Second, the device should be long enough for the mixing process to fully develop. Our work suggests that thirty times the transverse dimension is acceptable. Finally, the FRR must be large enough to maintain the medium polarity close to water, without operating with high dilution factors, which would generate waste. In our system, we may propose an FRR on the order of 3.

## Conclusion

The paper analyzes, in some depth, the mixing process developing along a device incorporating a Y junction and four rings. In the LNP context, this is a first. The device we analyze belongs to the category of inertial micromixers, i.e., micromixers taking advantage of the action of inertial (Dean) vortices and, in the meantime, operating below turbulence onset. These mixers are well adapted to high throughputs, a key condition for envisaging mass production. Such throughputs cannot be achieved, for instance, with the herringbone micromixer. In this paper, we showed that time-dependent phenomena develop above a certain threshold, close to the numerical work of Minakov et al.^18^. Our analysis of the mixing process, along with LNP measurements, indicates that the optimal conditions for producing functional structures require flow rates substantially above the onset of time-dependent phenomena. The rationale is that, as established in the literature on chaos (see, for instance, Ref^19^), oscillatory eddies give rise to efficient mixing. We suggest that the criterion we propose here (operating above the onset of oscillatory flow-regimes) is general. It should be useful for designing high-throughput micromixers dedicated to LNP production.

## Data Availability

The datasets analyzed during the current study are available from the corresponding author on reasonable request.
